# 3D positioning scheme exploiting nano-scale IR-UWB orthogonal pulses

**DOI:** 10.1186/1556-276X-6-544

**Published:** 2011-10-04

**Authors:** Nammoon Kim, Youngok Kim

**Affiliations:** 1 Kwangwoon University, 26 Kwangwoon-gil, Nowon-Gu, Seoul, 139-701, South Korea

**Keywords:** 3D positioning, nano-scale pulse, UWB, orthogonality, impulse radio

## Abstract

In these days, the development of positioning technology for realizing ubiquitous environments has become one of the most important issues. The Global Positioning System (GPS) is a well-known positioning scheme, but it is not suitable for positioning in in-door/building environments because it is difficult to maintain line-of-sight condition between satellites and a GPS receiver. To such problem, various positioning methods such as RFID, WLAN, ZigBee, and Bluetooth have been developed for indoor positioning scheme. However, the majority of positioning schemes are focused on the two-dimension positioning even though three-dimension (3D) positioning information is more useful especially in indoor applications, such as smart space, U-health service, context aware service, etc. In this paper, a 3D positioning system based on mutually orthogonal nano-scale impulse radio ultra-wideband (IR-UWB) signals and cross array antenna is proposed. The proposed scheme uses nano-scale IR-UWB signals providing fine time resolution and high-resolution multiple signal specification algorithm for the time-of-arrival and the angle-of-arrival estimation. The performance is evaluated over various IEEE 802.15.4a channel models, and simulation results show the effectiveness of proposed scheme.

## Introduction

The extraction of interesting features or positioning information from target objectives has become increasingly popular and required for realizing intelligent environment services such as smart space, U-health service, context aware service, etc [1-3]. It is well known that the outdoor positioning system has shown a lot of progress with Global Positioning System (GPS), which is a navigation system based on satellite signals. However, this method is useful only in the line-of-sight condition between satellites and GPS receivers, i.e., outdoor environments. It is hard to get the positioning information by using the GPS in in-door/building environments, where most urban peoples are active and reside. Recently, the importance of indoor positioning technology has been gradually increased because of rescue operations and disaster prevention in underground shopping centers, factories, logistics centers, and so on. As indoor positioning method, various systems such as RFID, WLAN, ZigBee, and Bluetooth have been considered, but their positioning errors are several meters to tens of meters. Moreover, most positioning researches have been focused on two-dimension (2D) positioning even though three-dimension (3D) positioning information is more useful in indoor applications. In indoor environments, the time-of-arrival (TOA) and the angle-of-arrival (AOA) approaches are well-known scheme for a high-precision ranging purpose.

The former estimates the distance between a mobile system (MS) and a base station (BS) by estimating the time-of-flight of signal and it requires minimum three BSs for the 2D positioning, while the latter estimates the receiving angle of the signal and it requires minimum two BSs. Various super-resolution techniques, like multiple signal specification (MUSIC) [4], minimum norm [5], and total least square estimation of signal parameter via rotational invariance techniques [6], have been researched for achieving precise ranging and angle information against severe multipath fading channels. Among them, MUSIC is the most widely used algorithm based on eigenvalue decomposition of an array input correlation matrix due to its high-resolution capability, simplicity, and low computational complexity. In the mean time, the impulse radio ultra-wideband (IR-UWB) signal is based on the radiation of a train of extremely short pulses, typically in the range of nanoseconds and sub-nanoseconds, which results in fine time resolution for high-precision ranging performance. However, the different positioning performance is caused by what type of pulse shape. For instance, the interference problem between the pulses transmitted at the same time can be resolved by using the orthogonal pulses, and thereby the diversity gain can be achieved with multiple orthogonal pulses.

**Figure 1:**
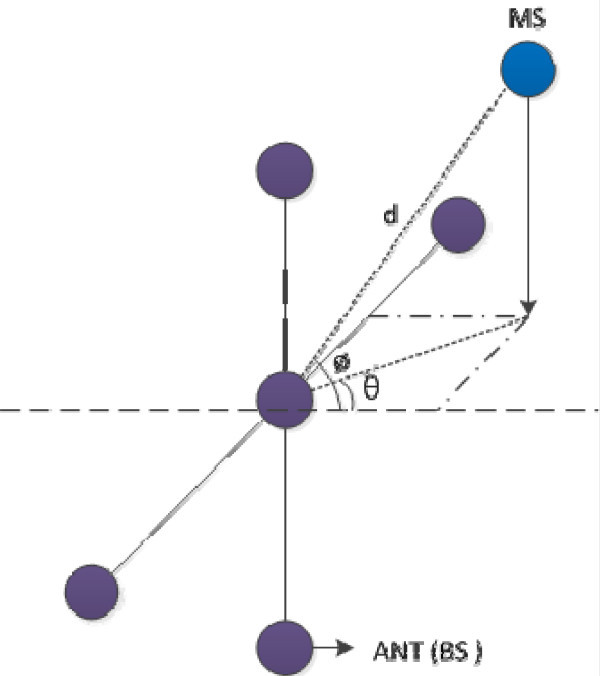
**Simplified schematic of 3D positioning scheme**. The elevation angle and the azimuth angle are estimated with vertical array antennas and horizontal array antennas, respectively, while the distance is estimated with centered antenna.

Since a hybrid TOA/AOA scheme can estimate the 2D position with only one BS, the 3D positioning system based on the hybrid TOA/AOA scheme is considered in this paper. Based on the hybrid scheme, the 3D positioning system with mutually orthogonal nano-scale IR-UWB signals and cross array antenna is proposed. Specifically, the proposed scheme uses nano-scale IR-UWB signals providing fine time resolution and the 2D MUSIC algorithm for estimating the TOA and the AOA simultaneously [7]. In the proposed scheme, elevation angle (*ø*), azimuth angle (*θ*), and distance (*d*) between MS and BS are estimated through cross array antennas and positioning algorithm. Figure 1 shows a simplified schematic of 3D positioning scheme. It is shown in the figure that the elevation and azimuth angles are respectively estimated with vertical array and horizontal array while the distance is estimated with centered antenna. The performance of proposed scheme is evaluated through the computer simulations over the IEEE 802.15.4a channel models (CMs) [8].

The rest of this paper is organized as follows. The proposed system description is addressed in "Background." In "Result," the effectiveness of the proposed approach is demonstrated with simulation results. The conclusion is made in "Conclusions."

## Background

### Orthogonal pulse

**Figure 2:**
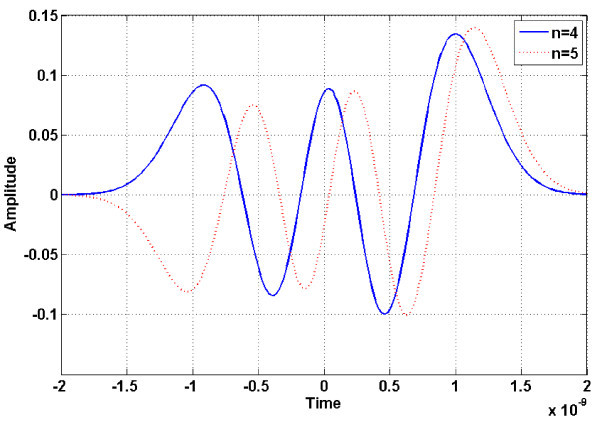
**Modified Hermite polynomial pulse shapes**. The fourth-order and fifth-order derivative MHP pulses satisfy the FCC indoor spectral mask and have orthogonal characteristic.

There have been many different types of the UWB pulses, e.g., Gaussian pulse, prolate spheroidal pulse, root raised cosine pulse, and modified Hermite polynomial (MHP) pulse. The cross-correlation property between orthogonal pulses is close to zero, and thus the orthogonal pulse is not only effective to prevent interference between uniform linear arrays (ULA) but also to achieve optimum performance based on channel characteristics. The orthogonal pulse can be used to improve performance of 3D positioning system. In this regard, we employ the MHP pulse, the most representative orthogonal pulse. The fourth-order and the fifth-order derivative MHP pulses, which are shown in Figure 2, satisfy the Federal Communication Commission (FCC) indoor spectral mask and have orthogonal characteristic. The MHP pulse is expressed as follows [9]:

(1) hn(t)=(-1)n exp[t24α]dndtn(exp[-t22]),

where *n *is order of the pulse and *α *is duration factor and its value is 1/128e17.

### System model

**Figure 3:**
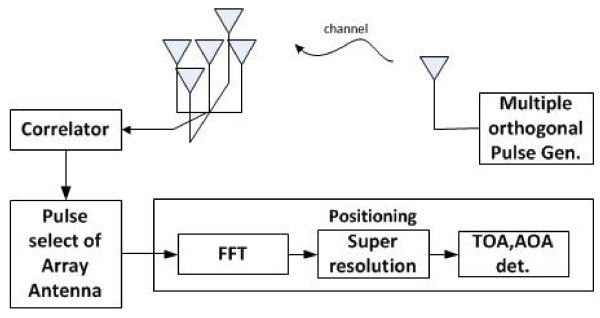
**Simplified proposed system structure**. The ULA receives the transmitted signal, and the detection with the high performance pulse shapes is evaluated with correlators. Based on the performance comparison for angle and distance estimations, position estimation can be performed by using MMSE and MUSIC algorithm with the selected pulse shape.

In the positioning system, the BS consists of two ULA with multiple antennas where the distance between two consecutive antennas is *d *= *c*/2*f_c_*, where *f_c _*represents the center frequency. Figure 3 shows a simplified proposed system structure for positioning where the ULA receives the signal from an MS and the phase difference among antennas is used to estimate the AOA. The arriving signal at each antenna is sampled into L samples, where the sampling interval is Δ*f *in frequency domain for TOA estimation. At the receiver side, the received signal over the multipath fading channel can be expressed as follows [10]:

(2) $$r_{u}\left(t\right)=\overset{K-1}{\underset{k=0}{\sum }}\alpha_{k}\beta_{u}\left(\theta_{k}\right)s\left(t-\tau_{k}\right)+w\left(t\right),$$

where *K *is the total number of multipath channels, *α_k _*is the amplitude, *β_u_*(*θ_k_*) is the response of the *u*th antenna to the *k*th path arriving from angle *θ_k_*, *τ*_*k *_is the propagation delay of the *k*th path, *S*(·) is the transmitted signal shape, and *w*(*t*) is the additive white Gaussian noise with mean zero and variance. By applying the harmonic signal model, Equation 2 can be rewritten in frequency domain as follow:

(3) $$\begin{array}{lll} R_{u}\left(f\right) & =\overset{K-1}{\underset{k=0}{\sum }}S\left(f\right)H\left(f\right)+W\left(f\right)\; & \text{(1)}\\ & =\overset{K-1}{\underset{k=0}{\sum }}\alpha_{k}S\left(f\right)e^{-j2\pi f\tau_{k}}e^{-j2\pi \theta_{k}}+W\left(f\right).\; & \text{(2)}\\ & \; & \text{(3)} \end{array}$$

The discrete measurement data of Equation 3 can be obtained by sampling at *L *equally spaced frequencies, and it is given as follows [7]:

(4) $$R_{u}\left(m\right)=\overset{K-1}{\underset{k=0}{\sum }}\overset{L-1}{\underset{l=0}{\sum }}\alpha_{k}S\left(m+l\right)e^{-j2\pi \left\{d\left(u-1\right)\frac{\sin\theta_{k}}{\lambda }+\left(f_{c}+l\Delta f\right)\tau_{k}\right\}}+W\left(m\right),$$

where *m *= 0, 1,..., *M *- 1. Since we use harmonic model for transmitted signal in *L *frequency samples, the *N *samples are divided into *M *consecutive segments of length *L*, where *M *= *N *- *L *+ 1. Thus, the transmitted signal *S *is formed into an *M *× *L *matrix and the sampled signal of Equation 4 can be rewritten as follows [4]:

(5) $$R_{u}=SH_{u}+W=S_{u}\alpha +W,$$

$$R={\left[R_{1}R_{2}\cdots R_{U}\right]}^{T}$$

where *U *is the number of antennas, and

$$S=\left[\begin{array}{cccc} S\left(0\right) & S\left(1\right) & \cdots \; & S\left(L-1\right)\\ \vdots & \vdots & \ddots & \vdots \\ S\left(M-1\right) & S\left(M\right) & \cdots \; & S\left(M+L-2\right) \end{array}\right]$$

$$V_{u}=\left[\begin{array}{cccc} v_{u,1}\left(\theta_{1},\tau_{1}\right) & v_{u,1}\left(\theta_{2},\tau_{2}\right) & \cdots \; & v_{u,1}\left(\theta_{K},\tau_{K}\right)\\ \vdots & \vdots & \ddots & \vdots \\ v_{u,L}\left(\theta_{1},\tau_{1}\right) & v_{u,L}\left(\theta_{2},\tau_{2}\right) & \cdots \; & v_{u,L}\left(\theta_{K},\tau_{K}\right) \end{array}\right]$$

$$v_{u,l}\left(\theta_{k},\tau_{k}\right)=e^{-j2\pi \left\{d\left(u-1\right)\frac{\sin\theta_{k}}{\lambda }+\left(l-1\right)\Delta f\tau_{k}\right\}}$$

$$\alpha =\text{diag}\left[\alpha_{1},\cdots \;,\alpha_{k},\cdots \;,\alpha_{K}\right]$$

$$W=\left[\begin{array}{cccc} w\left(1,1\right) & w\left(1,2\right) & \cdots \; & w\left(1,K\right)\\ \vdots & \vdots & \ddots & \vdots \\ w\left(M,1\right) & w\left(M,2\right) & \cdots \; & w\left(M,K\right) \end{array}\right]$$

The signal model in Equation 5 can be used to minimum mean square error (MMSE) for channel estimation and jointly estimate *θ_k _*and *τ_k _*in the 2D MUSIC algorithm. The detection with different pulse shapes is evaluated with correlators, and the estimation performance of angle and distance is compared at the receiver. Based on the performance comparison for angle and distance estimations, position estimation can be performed by selecting the pulse shape providing enhanced performance.

### Channel estimation

The channel impulse response (CIR) can be estimated by channel estimation methods such as zero forcing and MMSE. In this paper, MMSE is applied to the CIR. The CIR can be estimated by applying the inversion or pseudo-inversion of the known signal matrix. By multiplying both sides of Equation 5 by the inverse of the signal shape matrix *S*^+^, where $S^{+}=S^{H}{\left\{S\cdot S^{H}+\left(\sigma_{w}^{2}\right)\cdot I\right\}}^{-1}$, Equation 5 can be rewritten as follows:

(6) $$S^{+}R_{u}=S^{+}SH+S^{+}W\;\text{or}\;\tilde{H}=H+\tilde{W},$$

where *I *represents an identity matrix.

### Angle and distance estimation

AOA and TOA are jointly estimated by the MUSIC algorithm which has become a popular high-resolution method since it was pioneered by Schmidt. The algorithm is based on eigenvalue decomposition of correlation matrix, *R_XX_*. By finding the largest eigenvalues and eigenvectors (EVs), the signal can be distinguished from noise. For example, if the number of multipath signals is ***K***, the number of eigenvalues and eigenvectors is ***K***. The signal correlation matrix *R_XX _*can be expressed as follows:

(7) $$R_{XX}=E\left\{RR^{H}\right\}=SAS^{H}+\sigma_{w}^{2}I,$$

where *A *= *E *{*αα^H^*}. The correlation matrix *R_XX _*has the UL-dimensional subspace including two orthogonal subspaces of signal and noise. When the matrix *SAS^H^*, which corresponds to signal subspace, has the rank ***K***, the eigenvectors corresponding to ***K ***largest eigenvalues of *R_XX _*are called the signal EVs. On the other hand, the EVs corresponding to (UL-K) smallest eigenvalues of *R_XX _*are called the noise EVs. The signal and noise subspaces are spanned by the signal and noise eigenvectors, respectively. Because of the orthogonal condition between the signal and noise subspaces, the following pseudo-spectrum can estimate *θ_k _*and *τ_k _*for *k *= 1,..., ***K***:

(8) $$P_{\text{MUSIC}}\left(\theta ,\tau \right)=\frac{1}{\overset{\text{UL - K}}{\underset{i=1}{\sum }}{|s^{H}\left(\theta ,\tau \right)E_{i}|}^{2}},$$

where *E_i _*is the *i*th column vector of the noise eigenvectors and **s **= (*θ*, *τ*) is the column vector of *S *having arbitrary direction *θ *and delay, *τ*. For the special case that *θ *= *θ_k _*and *τ *= *τ_k_*, the corresponding signal vector is orthogonal to *E_i_*. Therefore, we can estimate the desired values by detecting the maximum value of the pseudo-spectrum on the AOA-TOA plane.

## Result

In this section, the performance of proposed scheme is evaluated through the computer simulations over the IEEE 802.15.4a CMs. For each ULA, the number of antennas is three. The pulse duration of IR-UWB signals is 2 ns. As channel models, the CM1, the CM3, and the CM5 of IEEE802.15.4a standard are employed for computer simulations. The parameters for simulations are set to *L *= 120, *K *= 57. It is assumed that the azimuth angle has a Laplacian distribution and the elevation angle has a Gaussian distribution. Variance of each angle is one and angle range is from -60 to 60 [11]. The characteristic of the channel model is shown Table 1.

**Table 1:** Characteristic of 802.15.4a channel models

Channel model	Center frequency (GHz)	Environment	MS and BS distance (m)
1	6	LOS residential	7 to 20
3	4.5	LOS office	3 to 28
5	4.5	LOS outdoor	5 to 17

**Figure 4:**
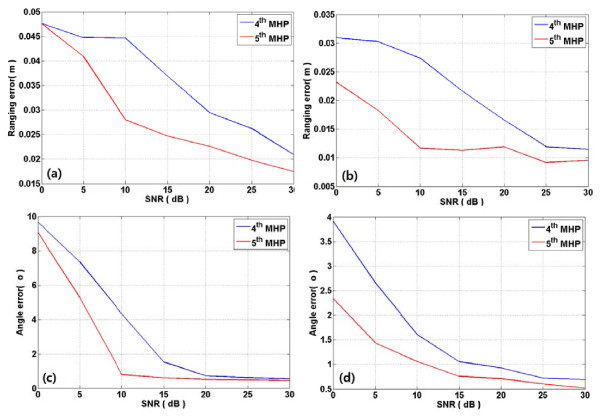
**Ranging and angle error in CM1 with fourth MHP and fifth MHP pulses**. Ranging and angle error in CM1. (**a**) and (**c**) are azimuth ranging error and angle error according to SNR. (**b**) and (**d**) are elevation ranging error and angle error according to SNR.

**Figure 5:**
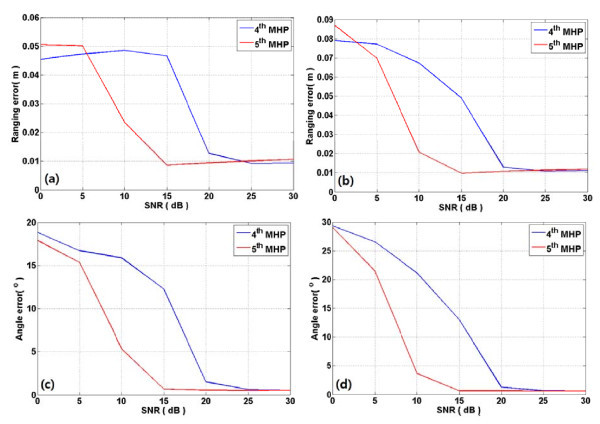
**Ranging and angle error in CM3 with fourth MHP and fifth MHP pulses**. Ranging and angle error in CM3. (**a**) and (**c**) are azimuth ranging error and angle error according to SNR. (**b**) and (**d**) are elevation ranging error and angle error according to SNR.

**Figure 6:**
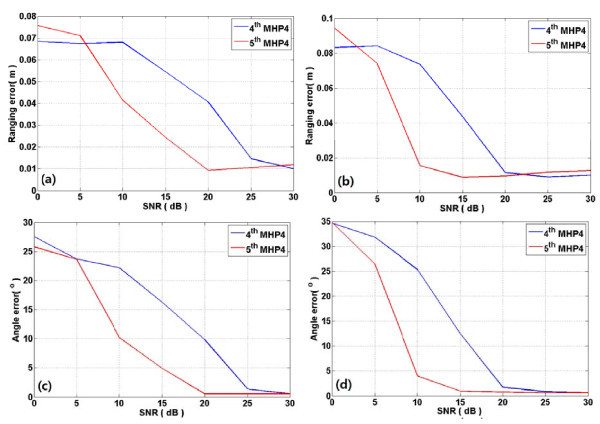
**Ranging and angle error in CM5 with fourth MHP and fifth MHP pulses**. Ranging and angle error in CM5. (**a**) and (**c**) are azimuth ranging error and angle error according to SNR. (**b**) and (**d**) are elevation ranging error and angle error according to SNR.

Figure 4 shows the ranging and angle estimation errors of TOA and AOAs from azimuth and elevation ULAs in CM1. As shown in the figure, the fifth MHP outperforms the fourth MHP over all signal-to-noise ratios (SNRs) with respect to ranging and angle errors. Figure 5 presents the ranging and angle estimation errors under same conditions in CM3. From this figure, the fifth MHP outperforms the fourth MHP in SNR from around 5 to 20 dB or so. On the other hand, the ranging performance of the fourth MHP outperforms that of the fifth MHP at other SNR levels. In case of angle error, the fifth MHP is superior to the fourth MHP in less than 25 dB, but two pulses show similar accuracy in more than 25 dB. Figure 6 depicts the ranging and angle estimation error of TOA and AOA of azimuth ULA and elevation ULA in CM5. As shown in the figure, the fourth MHP shows good ranging performance than fifth MHP in less than about 5 dB and in more than about 25 dB. In other part of the SNR, fifth MHP ranging performance is better than fourth MHP in both Azimuth and elevation. The fifth MHP outperform than fourth MHP in less than about 25 dB. In more than 25 dB, the performances of the two pulses are similar.

## Conclusions

In this paper, we evaluated performance of 3D positioning system with nano-scale IR-UWB pulses. In the CM1, fifth MHP confirmed that the performance is good than fourth MHP in all SNR. In the CM3 and the CM5, the fourth MHP showed good ranging performance in less than about 5 dB and in more than about 25 dB. The fifth MHP showed an excellent performance in angle estimation than fourth MHP in less than about 25 dB. However, the performances of the two pulses are similar in more than about 25 dB. The simulation results showed the different performance according to pulse shape and CMs. With consideration of SNR and channel environments, therefore, the use of different pulse shape can enhance estimation performance compared to that of the system using only one pulse.

## Competing interests

The authors declare that they have no competing interests.

## Authors' contributions

NK carried out the computer simulation. YK conceived and designed the 3D positioning system. All authors read and approved the final manuscript.
